# The transcriptome landscapes of allantochorion and vitelline-chorion in equine day 30 conceptus

**DOI:** 10.3389/fcell.2022.958205

**Published:** 2022-08-04

**Authors:** Yingchao Shen, Hong Ren, Toli Davshilt, Shuyue Tian, Xisheng Wang, Minna Yi, Tseweendolmaa Ulaangerel, Bei Li, Manglai Dugarjav, Gerelchimeg Bou

**Affiliations:** College of Animal Science, Inner Mongolia Key Laboratory of Equine Genetics, Breeding and Reproduction, Inner Mongolia Agricultural University, Hohhot, China

**Keywords:** allantochorion, vitelline-chorion, CG, RNA-seq, equine conceptus

## Abstract

During equine early gestation, trophectoderm forms chorion tissue, which is composed of two parts that one is covering allantoin, called allantochorion (AC) and another is covering yolk sac, which here we call vitelline-chorion (VC). Given that little is known about the equine trophoblast-derived chorion differentiation at an early stage, we first compared the transcriptome of AC and VC of day 30 equine conceptus based on RNA-sequencing. As a result, we found that compared to VC, there are 484 DEGs, including 305 up- and 179 down-regulated genes in AC. GO and KEGG analysis indicated that up-regulated genes in AC are mainly cell proliferation and cell adhesion-related genes, participating in allantois expansion and allantochorionic-placenta formation; dominant genes in VC are extracellular exosome and other cell adhesion-related genes implicated in direct and indirect conceptus-maternal communication. Additionally, as for the progenitor chorion tissue of equine chorionic gonadotropin secreting endometrium cup—the chorionic girdle (CG), which locates at the junction of the dilating AC and regressing VC, we revealed its unique gene expression pattern and the gene regulation during its further differentiation *in vitro*. Collectively, this study sheds light on the molecular events regarding the trophoblast differentiation and function at an early stage of the equine preimplantation conceptus.

## Introduction

Trophectoderm plays vital roles in embryonic development and implantation establishment mainly through its temporally and spatially regulated expression of adhesion and extracellular matrix (ECM) molecules that are crucial for blastocyst signaling, attachment, migration, and invasion (Haouzi et al., 2011). However, in addition to these common sides, the pattern of trophoblast early differentiation and function has shown highly placenta type- and species-specific properties (Carter and Enders, 2004). To better understand the exact role and functioning way of trophoblast, it is necessary to decipher its molecular events and regulatory networks.

Being one kind of epitheliochorial placenta type animal with a longer pre-implantation phase, as for the horse, trophectoderm participates in the embryo-maternal recognition, implantation, nourishment, and even pregnancy maintenance by secreting hormones like estrogen and equine chorionic gonadotropin (eCG) (Allen, 2001; Klein and Troedsson, 2011; Rivron et al., 2018; Smits et al., 2020). Moreover, the structure of the equine preimplantation conceptus is different from other mammals, especially after day 20, the allantois and yolk sac occupy the inner space of the globular embryo. When the main nutrition source will be changing from the yolk sac to the well-vascularized allantochorionic placenta, the allantois enlarges but the yolk sac regresses gradually (Allen and Stewart, 2001). On day 28–30 of gestation, a specialized band of trophoblast cells called the chorionic girdle (CG) becomes visible in the chorion at the junction of allantoic and yolk sac membranes. Hereafter, trophectoderm formed chorion could be easily dissected into allantochorion (AC) and chorion covering yolk sac (Vitelline-chorion, VC), because the CG keeps a boundary mark role along with yolk sac regression and allantois enlargement. Then on days 38–40, CG cells invade the uterine stroma to form endometrial cups and produce eCG (Hoppen, 1994). Both CG and endometrial cups are the structures unique to the equids.

In the last half-century, many researchers and teams have put great efforts into histologically and functionally exploring the equine trophoblast differentiation and laid the important foundation for the current equine reproduction knowledge (AO McKinnon et al., 2011; Aurich and Budik, 2015; Klein, 2016). However, it is widely admitted that compared to the studies in other domestic animals, molecular studies are still less in equids due to the late release of the equine genome and the difficulty of sampling. In recent years, high-throughput RNA sequencing was widely applied and mainly focused on ICM-TE to understand the equine blastocyst first lineage segregation (Klein and Troedsson, 2011; Iqbal et al., 2014; Klein, 2015; Smits et al., 2020), or invasive trophoblast cells CG (Cabrera-Sharp et al., 2014; Read et al., 2018) to explain CG differentiation and eCG secretion. However, it is still an open question prior to invasive CG differentiation, how does the chorion from the AC and the VC, which are of completely different fate and function after then. Hence, this study is mainly designed to explore the different expressional profiles in AC and VC via RNA-sequencing and better understand the trophoblast initial differentiation and functioning mechanism of different parts of the chorion.

## Material and methods

### Animals and sample preparation

All animal procedures were complete in accordance with the approval of the Inner Mongolia university ethics committee. The examination of follicle development, and cervical and uterine tone were carried out by daily ultrasonography and then artificial insemination (AI) was applied to the mares (Klein and Troedsson, 2011). After AI, mares were examined daily by rectal ultrasonography to observe the embryo development from 15 days after ovulation and to collect the embryo at 30 days after ovulation. A published non-surgically uterine lavage method (de Mestre et al., 2008) was used for embryo flushing. With the aid of dissecting microscope and ophthalmic instruments, the chorion parts separately outside the allantois and yolk sac were detached from the embryo, then washed with PBS and transferred to a centrifuge tube with 1 ml Trizol (ThermoFisher, USA), mixed will by pipette and snap-frozen in liquid nitrogen immediately for RNA-seq. Also, under a microscope, a prominent CG tissue can be easily dissected and isolated free of microorganism contamination. Based on our continuous observations, on day 30 of gestation, the CG locates at the equator and the equine conceptus is composed of an equal volume of allantois and yolk sac (the results shown in result 1).

### RNA sequencing

All sequencing work was performed by the Annoroad Gene Technology (Beijing, China). The smart-Seq2 method was used for first-strand cDNA synthesis to establish a sequencing library and an Oligo-dT primer was introduced to the reverse transcription reaction for first-strand cDNA synthesis, followed by PCR amplification to enrich the cDNA and magbeads purification step to clean up the production. Then the cDNA production was checked by Qubit^®^ 3.0 Flurometer and Agilent 2,100 Bioanalyzer to ensure the expected production with a length of around 1–2 kbp. Then the cDNA was sheared randomly by ultrasonic waves for Illumina library preparation protocol including DNA fragmentation, end-repair, 3’ ends A-tailing, adapter ligation, PCR amplification, and library validation. After library preparation, PerkinElmer LabChip^®^ GX Touch and Step OnePlus™ Real-Time PCR System were introduced for library quality inspection. Qualified libraries were then loaded on the Illumina Hiseq platform for PE150 sequencing.

### Bioinformatics analysis

The sequencing quality was assessed with FastQC and multiQC software and the clean reads were obtained by removing the adaptor sequences, reads with >5% ambiguous bases, and low-quality reads containing >20% bases with a Q-value < 20%. The clean data were analyzed by the established HISAT2-Stringtie-Ballgown method (Pertea et al., 2016). Gene expression levels were quantified by Fragments Per Kilobase of the transcript, per Million mapped reads (FPKM) with the in-house script, differential expressing genes (DEGs) were screened out under the criteria of |log2 fold change (FC)| > 1 and *p*-value < 0.05.

The Gene Ontology (GO) and Kyoto Encyclopedia of Genes and Genomes (KEGG) enrichment analyses of DEGs were performed based on the DAVID database, respectively, and the criteria for significance were set at a *p*-value of <0.05.

### qPCR assay

The tissues from the equine conceptus were collected with 1 ml QIAzol Lysis Reagent (Qiagen, GER) and total RNA was extracted and reverse transcribed to cDNA using the RNeasy Kits (Qiagen, GER) and High Capacity cDNA Reverse Transcription Kit (ThermoFisher, USA) according to the manufacturer’s instructions. Quality test of RNA and cDNA was tested by Epoch Microplate Spectrophotometer (Biotek, USA) and agarose gel electrophoresis.

The primers of reference gene GAPDH and target genes were designed using Primer5 software (Supplementary Table S1). Full-length mRNA sequences were downloaded from NCBI, and primers were synthesized by Sangon Biotech (Beijing, China). The gene expression was examined using the TB Green premix Ex Taq (Takata, JPN) according to the manufacturer’s instructions on a CFX96 PCR instrument (Biored, USA). Finally, the relative gene expression was calculated using the 2^−ΔΔCt^ method.

### Frozen section and immunofluorescence

The chorion tissue band including CG with its two sides 0.5 cm of AC and VC was obtained by the dissecting microscope and ophthalmic instruments, and soaked in 4% paraformaldehyde (Solarbio, CHN) for fixation. The optimum cutting temperature compound (SAKURA, JPN) is applied for embedding the whole tissue and meanwhile making the tissues spread and tiled using ophthalmic instruments. After solidification at -20°C, the tissue embedding blocks were continually sectioned with 7 μm thickness by freezing microtome. Being washed with PBST (0.1%Trion in PBS), sections were permeabilized and blocked in the solution (0.1% Triton-X 100, 5% donkey serum, and PBS) for 1 h at room temperature and incubated overnight at 4°C with primary antibodies: anti-ITGA11(Cat# DF8992), anti-CDH1(Cat# AF0131) and anti-PECAM1(Cat# AF6191) (1:200, Affinity Bioscience, CHN). Subsequently, the samples were washed three times with PBS, followed by incubation with Alexa Fluor 546 (1:1,000, ThermoFisher, USA) for 2 h at room temperature. Nuclei were counterstained with DAPI (Solarbio, CHN) for 10 min at room temperature, followed by PBS wash. At last, IF sections were observed with OLYMPUS FV3000 confocal microscope. The fluorescence intensity for red fluorescence of the three proteins was analyzed by ImageJ 1.52a.

### Cell culture

A previously established method was used for the equine CG cell culture (de Mestre et al., 2008). In brief, the CG tissue was isolated with the aid of a dissecting microscope, and placed into culture medium: DMEM (Gibco, USA) enriched with 100 U/ml penicillin-streptomycin (Gibco, USA), 0.5 mg/ml vitamin C and 0.4 mg/ml insulin (Sigma, USA). Girdle cells were gently scraped off from the basement membrane and underlying mesodermal cell layer using a scalpel blade, then transferred to the sterile tube with culture medium where they were allowed to settle on ice for 5 min. The lower layer of cells, which contained large clumps 1–2 mm in diameter, was then resuspended in culture medium with 10% FBS (Gibco, USA), and cultured at 37°C in 5% CO_2_.

## Results

### Preimplantation development of Mongolian horse

**FIGURE 1:**
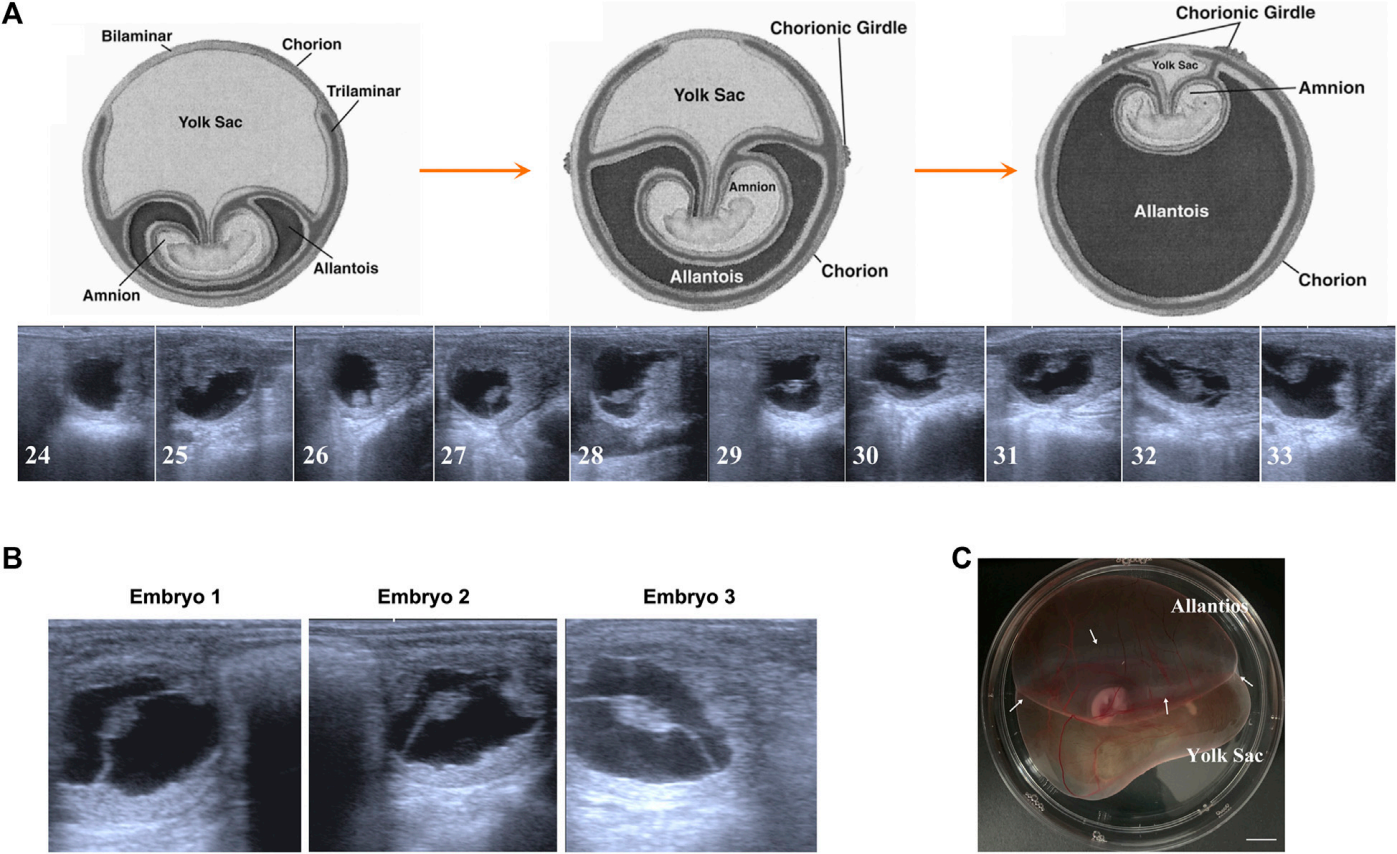
Early gestation development of Mongolian horse. **(A)** The ultrasonographic images of embryos from day 24 s to day 33. **(B)** The ultrasonographic image of day 30 embryos we used in this study. **(C)** Photo of day 30 equine embryo, white arrows show the chorion girdle, bar = 1 cm.

To our knowledge, the majority of fundamental data about equine embryonic development comes from some breeds from Europe or America. Therefore, first of all, to facilitate our further studies, we tracked the day 24 to day 33 preimplantation development of Mongolian horses using transrectal ultrasonography. As shown in Figure 1A, both embryo proper and the allantois enlarge during this period and on day 29–30, developing allantois and regressing yolk sac equally comprises half of the total volume of the conceptus. This developmental speed seems faster that the data in the former study on the western breed (van Niekerk and Allen, 1975). For Mongolian horses, on day 30 of gestation, the allantois and yolk sac have similar superficial areas on the globular conceptus, and at this time AC and VC are clearly bounded by a pale white CG band. Therefore, based on the Mongolian horse embryonic development and our purpose of research, we selected 3 day 30 conceptus with a prominent CG band around the equator line of conceptus to carry out the following studies (Figures 1B, C).

### Transcriptome comparison of equine allantochorion and vitelline-chorion

**FIGURE 2:**
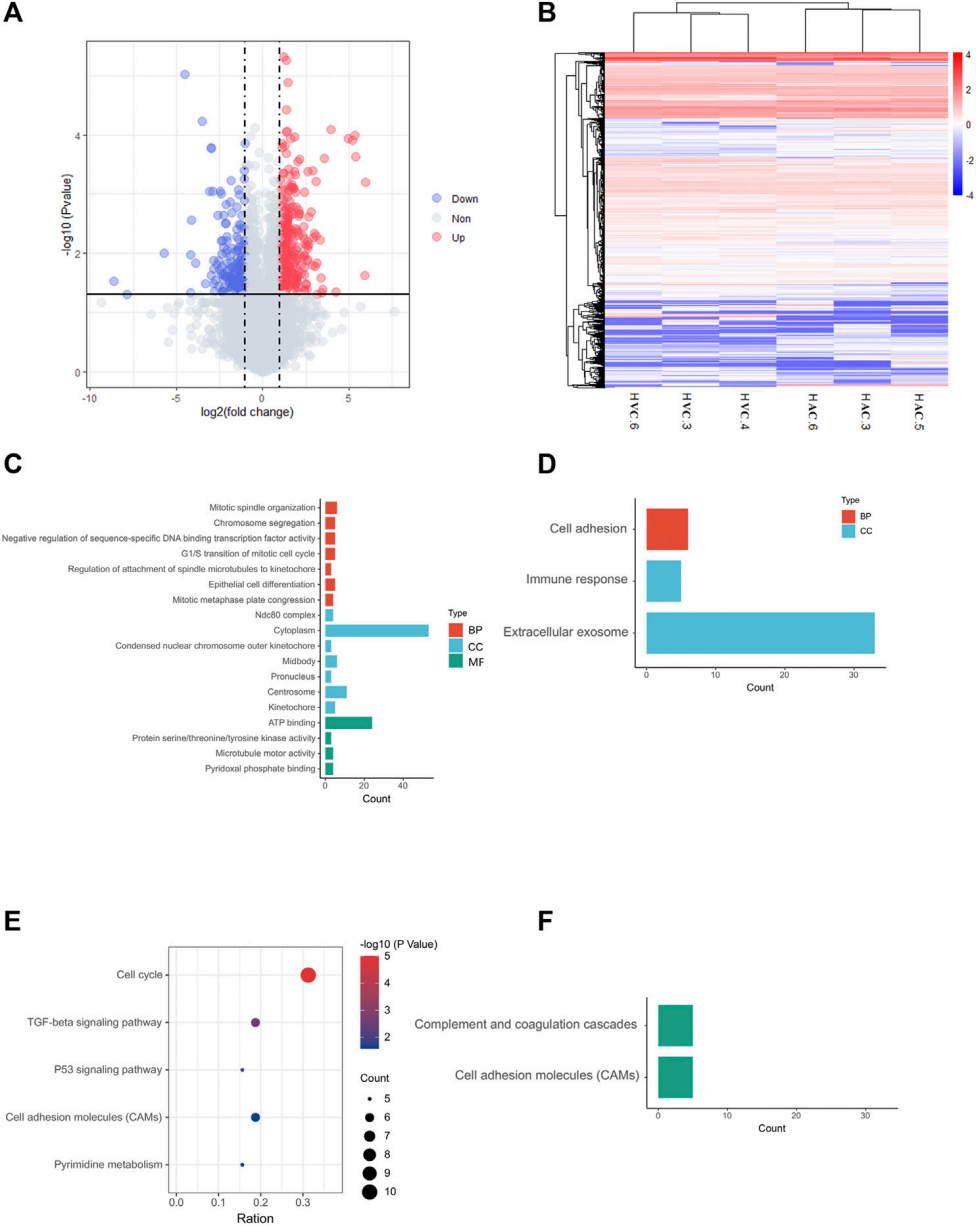
Results of RNA-seq and bioinformatic analysis. **(A)** Volcano map of DEGs in allantochorion (AC) virus chorion covering yolk sac (Vitelline-chorion, VC). The significantly upregulated and downregulated genes are shown as red and blue dots respectively. Gray dots indicate genes without significant differences. **(B)** Hierarchical cluster analysis of DEGs. Overall FPKM hierarchical clustering diagram is based on the log10 (FPKM +0.05) value for the normalization of the conversion (scale number) and clustering. Red represents high-expressed genes, and blue represents low-expressed genes. **(C, E)**, GO and KEGG analysis for up-regulated DEGs in AC selected on the basis of a *p*-value <0.05. **(D, F)**, GO and KEGG analysis for up-regulated DEGs in VC selected on the basis of a *p*-value <0.05.

In RNA sequencing of AC and VC, on average, 80 million clean reads were generated per sample, and in which 96% were mapped to the equine EquCab 3.0 reference genome. In total, 7,085 genes were identified after Ballgown-based filtering, and 305 up- and 179 down-regulated genes were identified in AC versus VC (Figure 2A, Supplementary Tables S2, S3).

The heat map shows that the AC and VC libraries had distinct patterns of gene expression, but the replicates within each group were similar (Figure 2B). GO and KEGG enrichment analyses were performed to further explore the function of the DEGs. The GO analysis of up-regulated genes in AC showed that they are mainly enriched in cell mitotic processes, such as mitotic spindle organization, G1/S transition of the mitotic cell cycle (Figure 2C), while the up-regulated genes in VC are only significantly enriched in cell adhesion, immune response and extracellular exosome (Figure 2D). Moreover, KEGG analysis showed that AC up-regulated genes are enriched in the cell cycle, p53 signaling pathway, TGF-beta signaling pathway, cell adhesion molecules, and pyrimidine metabolism, indicating that the active proliferation of AC cells and its preparation for placentation at this time (Figure 2E), whereas VC up-regulated genes are enriched in complement and coagulation-cascades and cell adhesion molecules (Figure 2F).

To reveal common features of two parts of chorion at this stage, we identified high expressed genes (FPKM>40) in both AC and VC to perform GO and KEGG analysis, and found that a large number of genes are enriched in translation, ribosome, and protein processing, Leukocyte transendothelial migration, Antigen processing and presentation, and ECM-receptor interaction, such as *RPL4* (Ribosomal Protein L4), *HSPA9* (Heat Shock Protein Family A Member 9), *PDIA3* (Protein Disulfide Isomerase Family A Member 3), *CCT3* (Chaperonin Containing TCP1 Subunit 3), emphasizing the active role of the chorion in embryonic development and maternal-embryonic cross-talk during early gestation (Supplementary Table S4, Supplementary Figure S1).

### Cells proliferation and mucin formation are major molecular events in day 30 allantochorion

**FIGURE 3:**
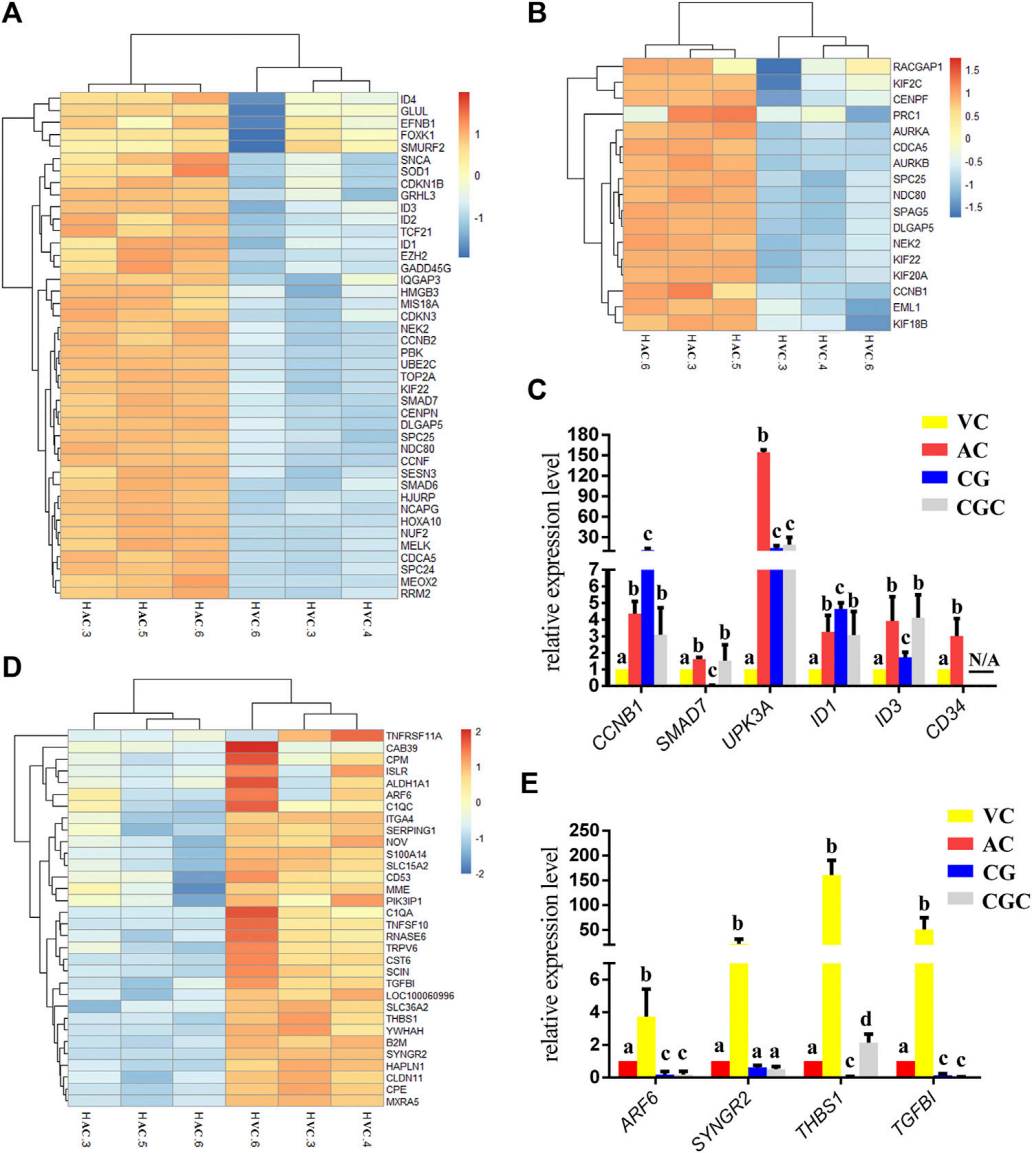
Heatmap of DEGs and histogram for qPCR results. **(A, B)**, Heatmap of DEGs up-regulated in allantochorion (AC): genes related to nuclear and chromosome activation in A, and genes related to spindle activation in B. **(C)** qPCR validation of DEGs up-regulated in AC. **(D)** Heat map representation of DEGs up-regulated in vitelline-chorion (VC). **(E)** qPCR validation of DEGs up-regulated in VC. Different letters in the histogram indicate a significant difference (*p*-value<0.05). CG, Chorionic girdle; CGC, Binuclear chorion girdle cells that formed after the *in vitro* culture of the chorion girdle.

Within the up-regulated genes in AC, we found that almost all genes were related to cell proliferation (Figure 2C), especially the genes related to the nucleus and chromosome activation (Figure 3A), and spindle activation (Figures 3B, C), including *CCNB1*(Cyclin B1), *SMAD7* (SMAD Family Member 7), *UPK3A* (Uroplakin 3A), and DNA-Binding Protein Inhibitors: *ID1*, *ID3*. It is reasonable to deduce that robust cell proliferation and efficient metabolism are major events for the expanding AC of day 30 conceptus. Moreover, pyridoxal, an important form of vitamin B6 in pregnancy, plays important role in the development of the placenta and fetus (Schenker et al., 1992)and has been proven related to mucin formation (Moran, 2017). In our results, we find that four genes which belong to the pyridoxal phosphate binding pathway, *ALAS2* (5′-Aminolevulinate Synthase 2), *DDC* (Dopa Decarboxylase), *BCAT1* (Branched Chain Amino Acid Transaminase 1), *GCAT* (Glycine C-Acetyltransferase), are highly expressed in day 30 AC (Figure 2C, Supplementary Table S2), implying the importance of vitamin B6 in AC development and equine placentation.

### Regressing chorion covering yolk sac plays a role in controlling equine embryo implantation

**FIGURE 4:**
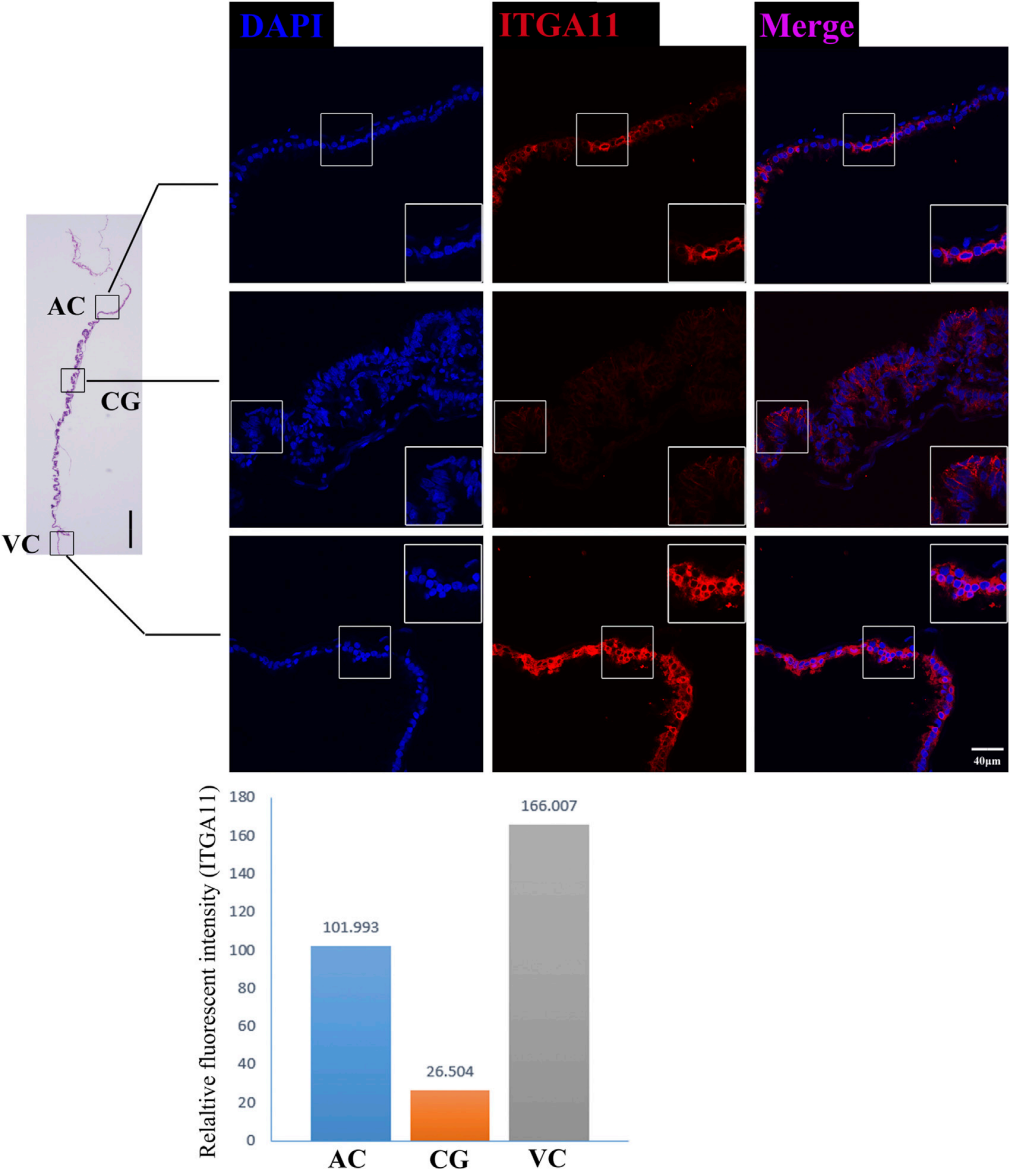
ITGA11 immunofluorescence on day 30 equine chorion. Histological images including allantochorion (AC), chorionic girdle (CG), and vitelline-chorion (VC) are used to clarify the location of the immunofluorescence view. Below, the histogram shows the fluorescence intensity for red fluorescence of ITGA11. An enlarged view of the area is shown in the white frame. Left bar = 400 μm, right bar = 40 μm.

On day 30, equine VC is still regressing. Inconsistent to it, not surprisingly we see that genes related to cell cycle and apoptosis regulation are highly expressed in it. However, the regressing VC seems not useless for equine conceptus. In our results, we also find that 31 DEGs related to implantation and cell adhesion, including *ARF6* (ADP Ribosylation Factor 6), *SYNGR2* (Synaptogyrin 2), *ISLR* (Immunoglobulin Superfamily Containing Leucine-Rich Repeat), and *ITGA11*, a key protein for collagen reorganization and cell adhesion (Lehnert et al., 1999), is robustly expressed in day 30 VC. What’s more, lots of DEGs are enriched in the functions and pathways associated with communication, such as extracellular exosome, immune response, and complement and coagulation cascades, indicating that the functionally active state of VC regarding the interaction with the maternal uterus environment (Figure 2D, Figures 3D, E, Figure 4).

For a day 30 equine conceptus heading to placentation, paradoxically, *TGFBI* (Transforming Growth Factor Beta Induced) known as a negative factor for cell attachment (Skonier et al., 1994), and *THBS1* (Thrombospondin-1), which can inhibit syncytiotrophoblast formation to trigger preeclampsia during placentation in human (Duan et al., 2021), is highly expressed in its VC (Figure 3E). Therefore, we propose that VC plays a more critical role in controlling implantation by expressing and orchestrating both positive and negative regulators for conceptus-maternal communication, cell adhesion, and migration.

### As the boundary of allantochorion and vitelline-chorion, the chorion girdle has its unique gene networks

**FIGURE 5:**
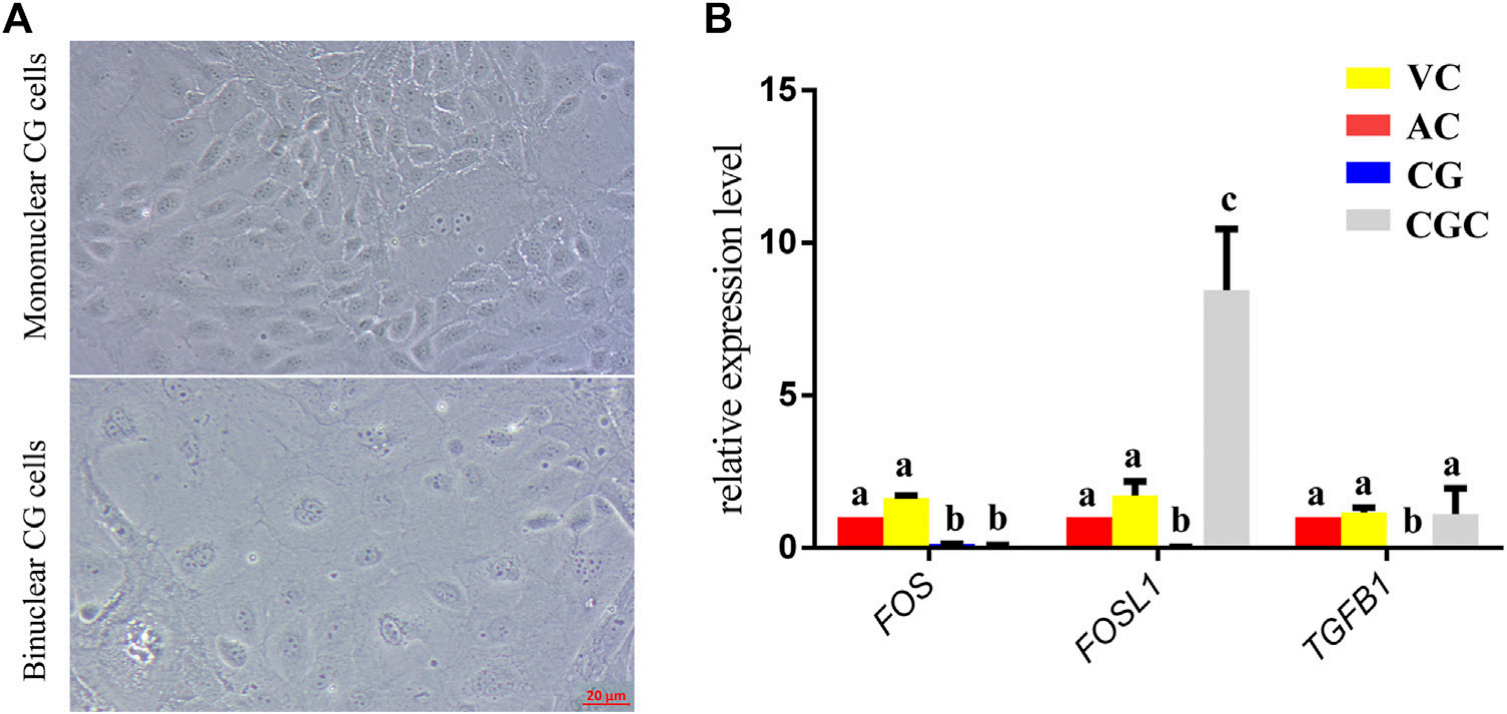
*In vitro* culture and gene expression of chorionic girdle (CG). **(A)** Morphology of chorionic girdle (CG) cells, the picture above is mononuclear CG cells, below is binuclear CG cells, bar = 20 μm. **(B)** qPCR results. Different letters indicate a significant difference (*p*-value<0.05).

**FIGURE 6:**
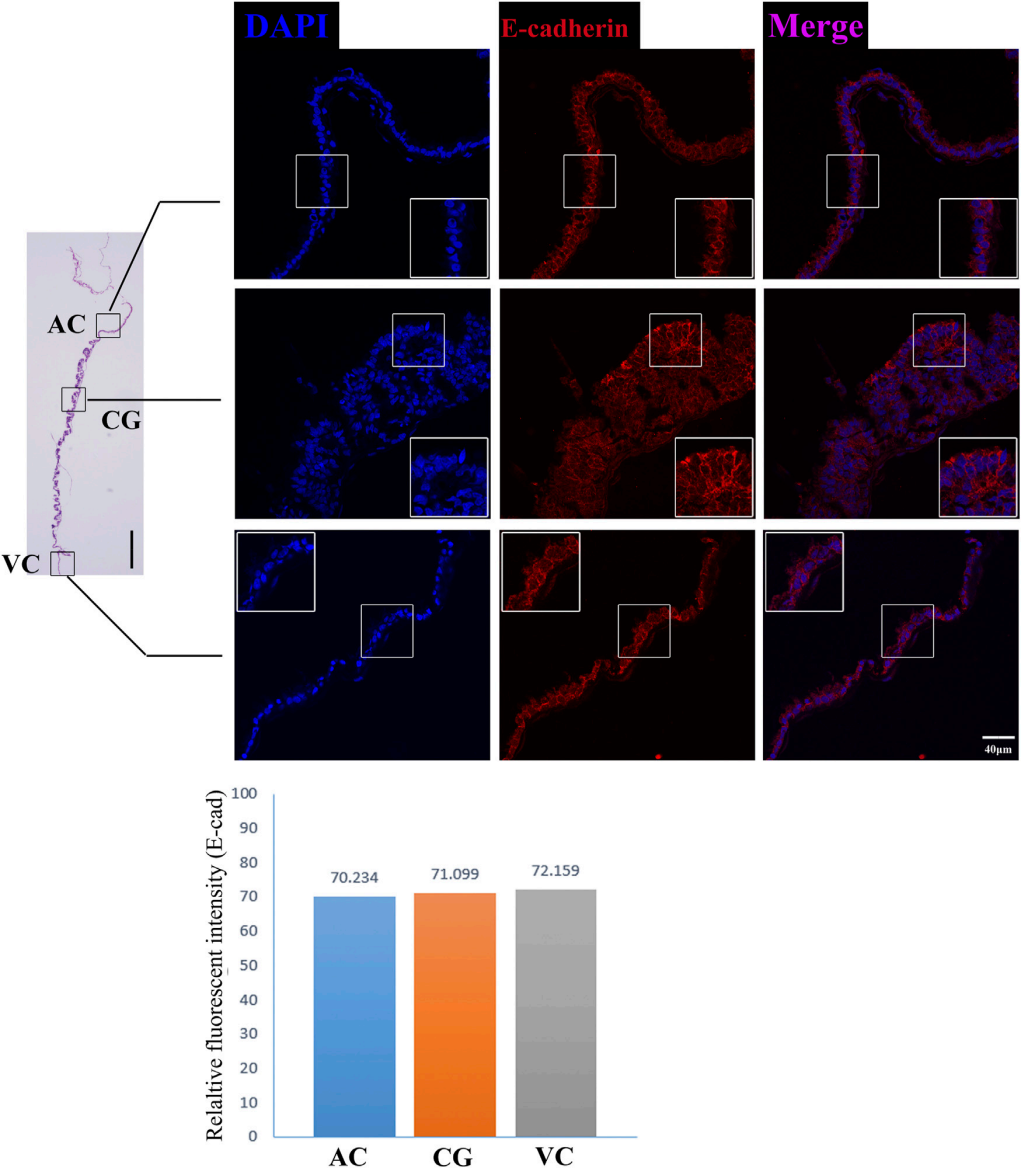
E-cadherin immunofluorescence on day 30 equine chorion., Histological images including allantochorion (AC), chorionic girdle (CG), and vitelline-chorion (VC) are used to clarify the location of the immunofluorescence view. Below, the histogram shows the fluorescence intensity for red fluorescence of E-cadherin. An enlarged view of the area is shown in the white frame. Left bar = 400 μm, right bar = 40 μm.

**FIGURE 7:**
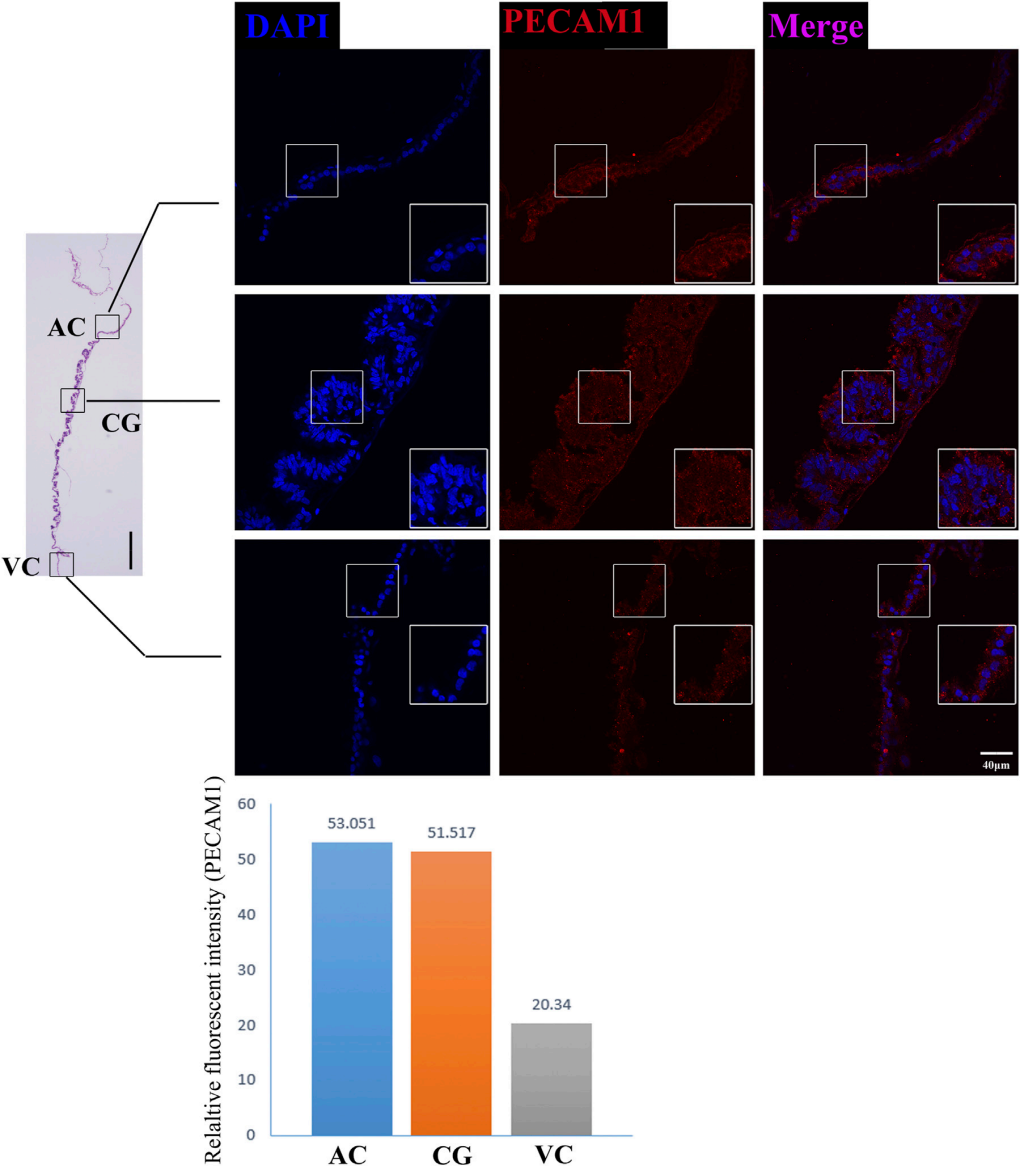
PECAM1 immunofluorescence on day 30 equine chorion. Histological images including allantochorion (AC), chorionic girdle (CG), and vitelline-chorion (VC) are used to clarify the location of the immunofluorescence view. Below, the histogram shows the fluorescence intensity for red fluorescence of PECAM1. An enlarged view of the area is shown in the white frame. Left bar = 400 μm, right bar = 40 μm.

The former studies have proved that CG not only is the borderline for AC and VC but also could differentiate towards the binuclear cells to form the endometrial cup and exert the function of eCG secretion and immunity after implantation (Antczak et al., 2013). *In vitro*, binuclear cells derive from the differentiation of the rapidly proliferating mononuclear CG cells (Figure 5A). Since little is known about the CG cell gene network, in this study we also simply compared the expression of some genes related to cell proliferation (Figure 3C), cell adhesion (Figure 3E), immune response, and trophoderm invasion (Figure 5B) in CG, binuclear CG cells, AC and VC by the method of qPCR. These results proved that CG may have unique gene sets different from AC and VC. Moreover, immunofluorescence assays showed although E-cadherin has the same expression levels in AC, VC, and CG (Figure 6), another two main implantation regulators *ITGA11* and *PECAM1* (Endothelial cell adhesion molecule 1) have distinct expression levels in CG (Figure 7, Supplementary Table S2, Supplementary Table S3). Moreover, the difference in gene expression between CG and the differentiated binuclear CG cells can be observed in the above qPCR results (Figures 3C, E, Figure 5B), indicating that the cytokine signaling pathway, FOS signaling pathway (Cui et al., 2019) and TGFβ signaling pathway (Kumar et al., 2015) are involved in CG cell differentiation.

## Discussion

This is the first study that investigated the gene profiling of each part of day 30 equine chorion. In spite of numerous substantial advances in equine reproduction, the molecular mechanisms underlying many stages of embryonic development are poorly understood. Here we mainly revealed that between AC and VC of day 30 equine embryos, there are 484 differential expressed genes. Among these, 305 genes are shown specifically up-regulated in enlarging AC, and it is not surprising that these genes are mainly related to cell mitosis regulation, for example, *NDC80*, *NUF2*, *SPC24*, and *SPC25*, which compose the nuclear division cycle 80 (Ndc80) complex to influence mitosis (Gao et al., 2020) and *CCNB1*, *EML1*, *AURKB*, *AURKA*, *SPC25*, which are primarily involved in mitotic spindle organization (Villegas et al., 2014; Cheng et al., 2021; Yin et al., 2021). These results could benefit our understanding of the physiological state of preimplantation AC, and the key genes and pathways controlling the AC enlargement.

AC is the main source of the equine fetal placenta (Franciolli et al., 2020), according to the previous study, its rapid proliferation and increasing interaction with endometrium are promoted by the uterus-derived hepatocyte growth factor-scatter factor (HGF-SF) and epidermal growth factor (EGF) (Lennard et al., 1998; Gerstenberg et al., 1999), and in accordance with it, we found AC has high-level expression of EGFR and HGFR. It is known that transforming growth factor-beta (TGF-B) plays an important role in embryo implantation (Maurya et al., 2013), consistently, the TFG-B pathway is significantly enriched in AC. In addition, the previous study indicated that ID proteins play critical roles in the regulation of endometrial epithelial cell function and conceptus development to support the establishment and maintenance of pregnancy in pigs (Han et al., 2018). Interestingly, our results showed that AC highly expresses *ID1*, *ID2*, *ID3,* and *ID4,* indicating the same bio function surrounding ID proteins may involve in the epitheliochorial placenta. It potentially provides an important reference for molecular diagnosis and exploration of chorionic development at equine early gestation.

Both AC-derived and endometrial mucin are needed for epitheliochorial placenta formation, here we found that plenty of genes on the vitamin B6 metabolism pathway are enriched in AC. The key role of vitamin B6 has been demonstrated in human pregnancy regarding the neurotransmitters and the placenta-supplying vitamin B6 is considered important for fetus amino acid metabolism (Bowling, 2011). Few data are available on the role of vitamins in equine embryogenesis and placentation, however, the enrichment of vitamin B6 metabolism-related genes in AC emphasizes the necessity of further exploration of the role of vitamin B6 in equine early gestation.

In contrast, we found that specific genes in regressing VC are much fewer than in AC. However, our data implied that although it is a regressing tissue, VC is still playing some critical roles before placentation. Except for the negative and positive cell adhesion regulator genes we have mentioned in our result part, another aspect we noticed is extracellular exosome-related genes. As we know, crosstalk between the endometrial epithelium and the embryo, especially the trophectoderm, is a prerequisite for successful implantation (Latifi et al., 2018), various molecular and functional changes occur to promote synchrony between the embryo and the endometrium as well as the uterine cavity microenvironment. As a homogenous population, Exosome originates from multivesicular bodies (MVBs) containing endosomes and can fuse with the plasma membrane and liberate the vesicles into the extracellular space (Antonyak and Cerione, 2015; Lo Cicero et al., 2015). There are growing evidence that exosomes derived from both uterus and embryos are involved in embryonic development, endometrial receptivity, and subsequent implantation (Thouas et al., 2015; Salamonsen et al., 2016). Charlotte et al. found that asynchrony equine embryo transfer resulted in large numbers of DEGs related to extracellular exosomes in endometrial and embryos (Gibson et al., 2020). Hence the VC-derived exosome probably plays an important role in conceptus-maternal communication and implantation. Further investigation on VC-derived exosomes would provide us with more clues on the cross-talk between the equine conceptus and the uterus.

Moreover, our RNA sequencing and immunofluorescence results together proved that chorion at this stage expresses a broad range of cell adhesion molecules such as *CDH5* (Brasch et al., 2011), *ALCAM* (Bowen et al., 1995), PECAM1(Dasgupta et al., 2009) and *CD34* (Fernandez-Vidal et al., 2006), and cell communication regulators *CLDN11*, *CADM3*, *CLDN3*, *ITGA4*, *ISLR*, *TGFBI*, *THBS1*, *HAPLN1*, but these molecules are differentially expressed in different parts of the chorion, emphasizing the work divisions as well as cooperation within chorion structures. Interestingly, we find some genes which negatively control cell adhesion and implantation are highly expressed in VC, so it is reasonable to propose that the VC plays vital roles in maternal-fetal recognition and the timing of implantation, like a rocket docking device with radar to ensure the conceptus successful implantation. These findings provide us with new insights into the mechanisms of the equine implantation process and diagnostic ideas for equine implantation failure.

In addition, we saw that, as the boundary structure in chorion at day 30, CG already possessed a unique gene expression network that is connected to the differentiation towards an invasive trophoblast part— eCG-secreting-endometrial cup cells. FOS and FOSL1 belong to the activating protein-1 (AP-1) transcription factor complex and are implicated in regulating gene networks controlling cellular invasion in diverse biological systems (Jiao et al., 2021). FOS and FOSL1 exert opposing effects on trophoblast invasion and FOSL1 plays a positive role in human trophoblast cells (Renaud et al., 2014). Inconsistent to it, in our results, we found that the expression of *FOSL1* in binuclear CG cells is higher than in AC, VC, and CG, but the expression of *FOS* in binuclear CG cells is lower than three parts of chorions. Therefore, we assumed that the AP-1 transcription factor complex may also play important roles in equine CG differentiation and invasion. Additionally, the transforming growth factor-beta (TGFB) family plays a role in controlling the trophoblast cells invasion (Lash et al., 2005), and the TGFB is activated in equine endometrial cups cells to maintain immune tolerance (Kammerer et al., 2020). In our result, we also found that the TGFB1 expression is significantly higher in binuclear CG cells. The importance of the TGFB gene family and FOS gene family in CG differentiation should be investigated in further study. eCG, also known as PMSG, plays an important role in animal reproduction and breeding, but at present, the only source we obtain this hormone is the blood of pregnant mares, which is hard to meet the animal welfare requirements (Manteca Vilanova et al., 2019). We believe that studies on the regulatory mechanism of CG cell differentiation would help us to explore a new approach to obtaining eCG from a cell-based system.

In summary, the transcriptome landscapes of allantochorion and vitelline-chorion in Mongolian horse day 30 embryos shown here shed light on the molecular events of different parts of the outermost conceptus membrane—chorion and the coordinatively ongoing epitheliochorial placentation processes.

## Data Availability

The datasets presented in this study can be found in online repositories. The names of the repository/repositories and accession number(s) can be found below: Bioproject, accession number: PRJNA844999.

## Supplementary material

Supplementary Figure S1 The GO and KEGG analysis for genes highly expressed in both allantochorion (AC) and vitelline-chorion (VC). The GO results are shown in regard to **(A)** Biological Process, **(B)** Cellular Component, and **(C)** Molecular Function. The KEGG results are shown in **(D)**. **(E)** Heatmap representation of genes that are related to protein processing in the endoplasmic reticulum and focal adhesion.
