# Supplementary material for: The transcriptome landscapes of allantochorion and vitelline-chorion in equine day 30 conceptus
Source: Front Cell Dev Biol. 2022 Aug 4;10:958205. doi: 10.3389/fcell.2022.958205 (PMC9386053; doi:10.3389/fcell.2022.958205)
Supplement: Supplementary file 3 [file Table1.DOCX]

| gene name | | primer（5‘-3’） |
| --- | --- | --- |
| CCNB1 | S | GCCTTCTCTGACGTAATTCTTGCAG |
|  | A | GGTCTGACTGATTGCTCTTCCTCA |
| SMAD7 | S | GCCTGTGTGTTGAGCTCTGCTGT |
|  | A | GGTGACGATGGACCGAATCCTT |
| UPK3A | S | GTTTGACAGCTCAGCAGCCCTCC |
|  | A | CTTGGTGCTGTCCTGTACAGAGGC |
| ID1 | S | CTACGACATGAACGGCTGCTACTCG |
|  | A | CAGCTCCAACTCCAGGTCCCAGAT |
| ID3 | S | GACAGTCCGCATCTTCCTATCCAG |
|  | A | GGTCAGTGGCAGAAACTCCTCTTG |
| CD34 | S | GCAGGTCAACATATTGGTCAAGC |
|  | A | GGTGCCAGTCTCATTAAGCCAAAG |
| ARF6 | S | GACAGGAACTGGTATGTGCAGCC |
|  | A | CCTATGAATGGTTCGCTCCTCTCC |
| SYNGR2 | S | GCTACAGCAATACCCACGAGTCC |
|  | A | CGACCACGAAGAAGAAGGCACAGG |
| THBS1 | S | GAACCACACCAGAAGACATCCTCAG |
|  | A | CTTGTGGCCGATGTAGTTAGTGC |
| TGFBI | S | GTTGGCGATGAAATCCTGGTTAG |
|  | A | CTGACTTCCAGCTTGTCACCTTG |
| FOS | S | CTGAGGCTGCCACCCCTGAATCT |
|  | A | CTGATGCTCTTGACAGGCTCCAC |
| FOSL1 | S | GTGCTAGAGAACTCCTGCAAGCC |
|  | A | CAGAGTTGGGTGGATCATAGGAAGAG |
| TGFB1 | S | GCTGTCCTTTGATGTCACCGGA |
|  | A | CCCACGCGGAGTGTGTTATCTT |
