# Supplementary figures and images for: The transcriptome landscapes of allantochorion and vitelline-chorion in equine day 30 conceptus
Source: Front Cell Dev Biol. 2022 Aug 4;10:958205. doi: 10.3389/fcell.2022.958205 (PMC9386053; doi:10.3389/fcell.2022.958205)

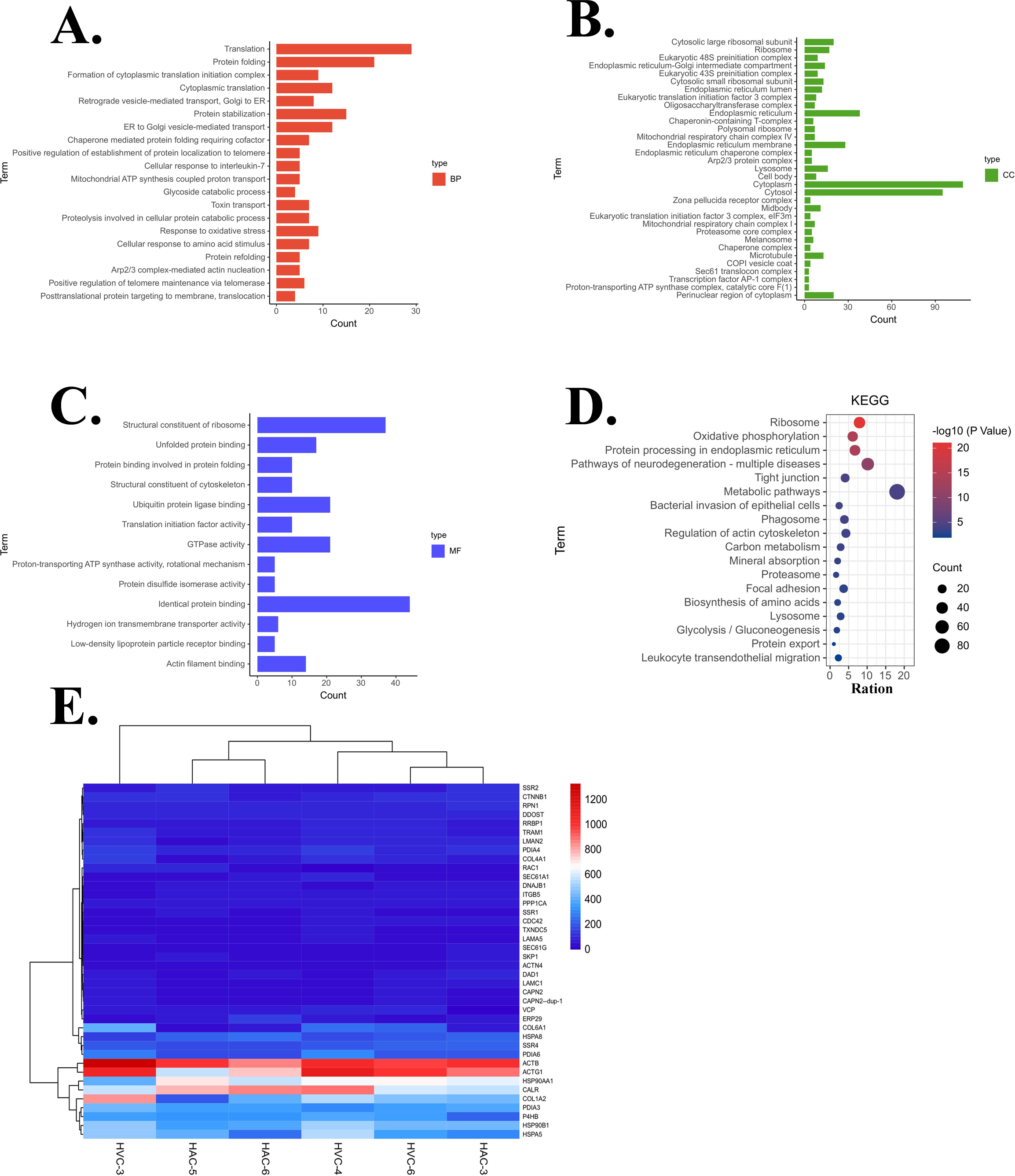

Supplement: Supplementary file 4 [file Figure8.TIF]
